# Community tuberculosis screening, testing and care, Uganda

**DOI:** 10.2471/BLT.23.290641

**Published:** 2024-04-30

**Authors:** Stavia Turyahabwe, Muzamiru Bamuloba, Levicatus Mugenyi, Geoffrey Amanya, Raymond Byaruhanga, Joseph Fry Imoko, Mabel Nakawooya, Simon Walusimbi, Jasper Nidoi, Aldomoro Burua, Moorine Sekadde, Winters Muttamba, Moses Arinaitwe, Luzze Henry, Rose Kengonzi, Mary Mudiope, Bruce J Kirenga

**Affiliations:** a National TB and Leprosy Program, Ministry of Health, Uganda, 6 Lourdel Road, Wandegeya, Kampala, Uganda.; b Department of Research and Innovation, Makerere University Lung Institute, Kampala, Uganda.; c Department of Statistics, The Medical Research Council/Uganda Virus Research Institute and London School of Hygiene & Tropical Medicine Uganda Research Unit, Entebbe, Uganda.; d Department of Health Systems Strengthening, Infectious Diseases Institute, Kampala, Uganda.

## Abstract

**Objective:**

To assess the effectiveness of a community-based tuberculosis and leprosy intervention in which village health teams and health workers conduct door-to-door tuberculosis screening, targeted screenings and contact tracing.

**Methods:**

We conducted a before-and-after implementation study in Uganda to assess the effectiveness of the community tuberculosis intervention by looking at reach, outputs, adoption and effectiveness of the intervention. Campaign 1 was conducted in March 2022 and campaign 2 in September 2022. We calculated percentages of targets achieved and compared case notification rates during the intervention with corresponding quarters in the previous year. We also assessed the leprosy screening.

**Findings:**

Over 5 days, campaign 1 screened 1 289 213 people (2.9% of the general population), of whom 179 144 (13.9%) fulfilled the presumptive tuberculosis criteria, and 4043 (2.3%) were diagnosed with bacteriologically-confirmed tuberculosis; 3710 (91.8%) individuals were linked to care. In campaign 2, 5 134 056 people (11.6% of the general population) were screened, detecting 428 444 (8.3%) presumptive tuberculosis patients and 8121 (1.9%) bacteriologically-confirmed tuberculosis patients; 5942 individuals (87.1%) were linked to care. The case notification rate increased from 48.1 to 59.5 per 100 000 population in campaign 1, with a case notification rate ratio of 1.24 (95% confidence interval, CI: 1.22–1.26). In campaign 2, the case notification rate increased from 45.0 to 71.6 per 100 000 population, with a case notification rate ratio of 1.59 (95% CI: 1.56–1.62). Of the 176 patients identified with leprosy, 137 (77.8%) initiated treatment.

**Conclusion:**

This community tuberculosis screening initiative is effective. However, continuous monitoring and adaptations are needed to overcome context-specific implementation challenges.

## Résumé

**Objectif:**

Évaluer l'efficacité d'une intervention de lutte contre la tuberculose et la lèpre au niveau communautaire, dans le cadre de laquelle des équipes et agents de santé du village effectuent un dépistage en porte-à-porte, des analyses ciblées et un suivi des contacts.

**Méthodes:**

Nous avons mené une étude en Ouganda, avant et après la mise en œuvre, afin de mesurer les performances de cette intervention communautaire en examinant sa portée, ses résultats, son adoption et son efficacité. La campagne 1 s'est déroulée en mars 2022 et la campagne 2 en septembre 2022. Nous avons calculé les pourcentages d'objectifs atteints et comparé le taux de signalement des cas durant l'intervention avec les trimestres correspondants de l'année précédente. Nous avons également évalué le dépistage de la lèpre.

**Résultats:**

En l'espace de cinq jours, la campagne 1 a passé en revue 1 289 213 personnes (2,9% de la population générale); parmi elles, 179 144 (13,9%) remplissaient les critères d'une tuberculose présumée, 4043 (2,3%) ont reçu un diagnostic de tuberculose avec confirmation bactériologique et 3710 (91,8%) ont fait l'objet d'un suivi médical. Lors de la campagne 2, 5 134 056 personnes (11,6% de la population générale) ont fait l’objet d’un dépistage; 428 444 (8,3%) cas présumés de tuberculose ont été détectés et 8121 (1,9%) d'entre eux ont reçu une confirmation bactériologique, tandis que 5942 individus (87,1%) ont fait l'objet d'un suivi médical. De son côté, le taux de signalement des cas a augmenté, passant de 48,1 à 59,5 par 100 000 habitants durant la campagne 1, ce qui équivaut à un rapport de taux de 1,24 (intervalle de confiance (IC) de 95%: 1,22-1,26). Et durant la campagne 2, le taux de signalement des cas a progressé de 45,0 à 71,6 par 100 000 habitants, avec un rapport de taux de 1,59 (IC de 95%: 1,56-1,62). Sur les 176 patients caractérisés par un diagnostic de lèpre, 137 (77,8%) ont entamé un traitement.

**Conclusion:**

Cette initiative de dépistage communautaire s'est révélée efficace. Il est cependant nécessaire d'exercer une surveillance continue et de procéder à des adaptations pour surmonter les difficultés de mise en œuvre liées au contexte.

## Resumen

**Objetivo:**

Evaluar la eficacia de una intervención comunitaria contra la tuberculosis y la lepra en la que los equipos y los profesionales sanitarios de los pueblos realizan pruebas de detección de la tuberculosis puerta a puerta, pruebas de detección selectivas y rastreo de contactos.

**Métodos:**

Se realizó un estudio de implementación antes y después en Uganda para evaluar la eficacia de la intervención comunitaria contra la tuberculosis mediante el análisis del alcance, los resultados, la adopción y la eficacia de la intervención. La campaña 1 se llevó a cabo en marzo de 2022 y la campaña 2 en septiembre de 2022. Se calcularon los porcentajes de objetivos alcanzados y se compararon las tasas de notificación de casos durante la intervención con los trimestres correspondientes del año anterior.Además, se evaluó la detección de la lepra.

**Resultados:**

Durante cinco días, en la campaña 1, se sometió a cribado a 1 289 213 personas (el 2,9% de la población general), de las cuales 179 144 (el 13,9%) cumplían los criterios de presunción de tuberculosis y a 4043 (el 2,3%) se les diagnosticó tuberculosis confirmada bacteriológicamente; 3710 (el 91,8%) personas recibieron asistencia. En la campaña 2, se sometió a cribado a 5 134 056 personas (11,6% de la población general), detectándose 428 444 (8,3%) pacientes con tuberculosis presunta y 8121 (1,9%) con tuberculosis confirmada bacteriológicamente; 5942 personas (87,1%) recibieron asistencia. La tasa de notificación de casos aumentó de 48,1 a 59,5 por 100 000 habitantes en la campaña 1, con una razón de tasa de notificación de casos de 1,24 (intervalo de confianza del 95%, IC: 1,22-1,26). En la campaña 2, la tasa de notificación de casos aumentó de 45,0 a 71,6 por 100 000 habitantes, con una razón de tasa de notificación de casos de 1,59 (IC del 95%: 1,56-1,62). De los 176 pacientes identificados con lepra, 137 (77,8%) iniciaron tratamiento.

**Conclusión:**

Esta iniciativa comunitaria de detección de la tuberculosis es eficaz. Sin embargo, se necesita un seguimiento continuo y adaptaciones para superar los desafíos de implementación específicos del contexto.

## ملخص

**الغرض:**

تقييم فعالية التدخل المجتمعي لمكافحة السل والجذام، حيث تقوم الفرق الصحية القروية والعاملون في المجال الصحي بإجراء فحص للسل من خلال الزيارات المنزلية، والفحوصات التي تستهدف النقاط الساخنة، وتتبع المخالطين.

**الطريقة:**

قمنا بإجراء دراسة قبل التنفيذ وبعده في أوغندا لتقييم فعالية التدخل المجتمعي لمكافحة السل من خلال النظر في مدى وصول التدخل، ومخرجاته، واعتماده، وفعاليته. تم إجراء الحملة الأولى في مارس/آذار 2022، والحملة الثانية في سبتمبر/أيلول 2022. وقمنا بحساب النسب المئوية للأهداف التي تم تحقيقها، ومقارنة معدلات الإبلاغ عن الحالات أثناء التدخل مع الأرباع السنوية المقابلة في العام السابق. وقمنا أيضًا بتقييم فحص الجذام.

**النتائج:**

طوال 5 أيام، قامت الحملة الأولى بفحص 1289213 شخصًا (%2.9 من إجمالي السكان)، منهم 179144 (%13.9) مستوفين لمعايير السل المفترضة، وتم تشخيص 4043 (%2.3) شخصًا كمصابين بالسل المؤكد جرثوميًا، و3710 شخصًا (%91.8) تم ربطهم بالرعاية. في الحملة الثانية، تم فحص 5134056 شخصًا (%11.6 من إجمالي السكان)، وتم اكتشاف 428444 (%8.3) من مرضى السل المفترضين، و8121 (%1.9) من مرضى السل المؤكدين جرثوميًا؛ و5942 شخصًا (%87.1) تم ربطهم بالرعاية. ارتفع معدل التبليغ عن الحالات من 48.1 إلى 59.5 لكل 100000 من السكان في الحملة الأولى، مع نسبة لمعدل الإبلاغ عن الحالات قدرها 1.24 (بفاصل ثقة مقداره %95: 1.22 إلى 1.26). في الحملة الثانية، ارتفع معدل الإبلاغ عن الحالات من 45.0 إلى 71.6 لكل 100000 من السكان، مع نسبة لمعدل الإبلاغ عن الحالات قدره 1.59 (بفاصل ثقة مقداره %95: 1.56 إلى 1.62). من بين 176 مريضًا تم تشخيص إصابتهم بالجذام، 137 مريضًا (77.8%) بدء علاجهم.

**الاستنتاج:**

تتميز مبادرة فحص السل المجتمعية هذه بالفعالية. ومع ذلك، فإن هناك حاجة إلى الرصد المستمر وعمليات التكيف للتغلب على تحديات التنفيذ الخاصة بالأوضاع.

## 摘要

**目的:**

评估基于社区实施结核病和麻风病干预措施的有效性，在该干预措施实施过程中，村卫生队和卫生工作者挨家挨户的进行结核病筛查、进行有针对性的筛查并追踪接触者。

**方法:**

我们在乌干达进行了一项实施前后对比研究，通过考虑干预措施的覆盖范围、实施情况、采纳率和有效性来评估基于社区实施的结核病干预措施的有效性。第 1 轮筛查于 2022 年 3 月进行，第 2 轮筛查于 2022 年 9 月进行。我们计算了目标实现的百分比，并将实施干预措施期间的病例通报率与前一年同季度的数据进行了比较。我们还评估了麻风病筛查

**结果:**

在 5 天的时间里，第一轮筛查覆盖了 1,289,213 人（占总人口的 2.9%），其中 179,144 人 (13.9%) 被初步推断为患有结核病，4,043 人 (2.3%) 被诊断为患有菌阳结核病；3,710 人 (91.8%) 需接受保健护理。第二轮筛查覆盖了 5,134,056 人（占总人口的 11.6%），其中 428,444 人 (8.3%) 被初步推断为患有结核病，8,121 人 (1.9%) 被诊断为患有菌阳结核病；5,942 人 (87.1%) 需接受保健护理。在第一轮筛查中，病例通报率从每 10 万人 48.1 例增加到了 59.5 例，病例通报率比值为 1.24（95% 置信区间，CI：1.22-1.26）。在第二轮筛查中，病例通报率从每 10 万人 45.0 例增加到了 71.6 例，病例通报率比值为 1.59（95% CI：1.56-1.62）。在 176 例确诊的麻风病患者中，有137例 (77.8%)开始了治疗。

**结论:**

此项基于社区实施的结核病筛查举措是有效的。但仍需持续的监测和调整干预措施，以应对在具体实施过程中突然出现的挑战。

## Резюме

**Цель:**

Оценить эффективность вмешательства по борьбе с туберкулезом и лепрой по месту жительства, в рамках которого сельские бригады медицинской помощи и медико-санитарные работники проводят скрининг на туберкулез на дому, целевые скрининги и отслеживание контактов.

**Методы:**

Для оценки эффективности вмешательства по борьбе с туберкулезом на уровне общин было проведено исследование реализации проекта «до и после» в Уганде, в ходе которого изучались охват, результаты, внедрение и эффективность вмешательства. Кампания 1 была проведена в марте 2022 года, кампания 2 – в сентябре 2022 года. Было подсчитано процентное соотношение достигнутых целей, а также проведено сравнение показателей уведомлений о случаях заболевания в период вмешательства с соответствующими показателями за кварталы предыдущего года. Также была проведена оценка скрининга на лепру.

**Результаты:**

За 5 дней в рамках кампании 1 было обследовано 1 289 213 человек (2,9% от общей численности населения), из которых 179 144 (13,9%) соответствовали критериям предположительного заболевания туберкулезом, у 4043 (2,3%) был диагностирован бактериологически подтвержденный туберкулез, а 3710 человек (91,8%) были направлены на лечение. В ходе кампании 2 было обследовано 5 134 056 человек (11,6% от общей численности населения), выявлено 428 444 (8,3%) предположительно больных туберкулезом и 8121 (1,9%) бактериологически подтвержденный больной туберкулезом, 5942 человека (87,1%) были направлены на лечение. В ходе кампании 1 показатель заболеваемости увеличился с 48,1 до 59,5 на 100 000 населения, при этом показатель регистрируемой заболеваемости составил 1,24 (девяностопятипроцентный доверительный интервал, 95%-й ДИ: 1,22–1,26). В ходе кампании 2 частота выявления случаев увеличилась с 45,0 до 71,6 на 100 000 населения, при этом показатель регистрируемой заболеваемости составил 1,59 (95%-й ДИ: 1,56–1,62). 137 (77,8%) из 176 пациентов, у которых была обнаружена лепра, начали лечение.

**Вывод:**

Эта инициатива по проведению скрининга на туберкулез в общинах является эффективной. Однако для решения проблем реализации в конкретных условиях необходимы постоянный мониторинг и адаптация.

## Introduction

Tuberculosis remains one of the leading causes of death worldwide.^1^ In 2022, approximately 10.6 million people fell sick with tuberculosis globally, with most cases occurring in South-East Asia (45%) and African (23%) regions.^1^ Various national tuberculosis programmes have relied on passive case finding to find tuberculosis cases, but this can lead to high rates of delayed or missed (undiagnosed or unreported) cases.^2^^–^^4^ Active case finding in the community has emerged as a more effective approach, as it detects tuberculosis at early stages of disease and has the potential to be cost effective among populations with a high burden of tuberculosis.^4^^–^^7^ For example, in Ethiopia a case finding intervention that consisted of advocacy, training, stakeholder engagement and active case finding by health extension workers led to an increase in both case notification and treatment success.^8^ Similar interventions have demonstrated feasibility, acceptability and impact in addressing tuberculosis epidemiology; these have led to reduced tuberculosis prevalence, increased notifications, and improved treatment outcomes.^9^^–^^11^

Uganda is a country with a high tuberculosis burden; its incidence has remained fairly constant over the years at around 198 cases per 100 000 population per year, with a high proportion of missed tuberculosis cases.^12^ The case notification rate had been declining until 2018, at which point the national tuberculosis programme implemented active case finding strategies. Case notifications subsequently improved but, during the coronavirus disease 2019 (COVID-19) pandemic, notifications declined by 7.5% (dropping from 65 857 cases in 2019 to 60 887 in 2020).^13^^,^^14^

To address the issue of missed tuberculosis cases in Uganda, in 2022, its national tuberculosis programme embarked on a new country-wide, community-based, active case finding campaign dubbed CAST TB, with the focus on community awareness, screening, testing, prevention, and treatment to end tuberculosis (hereafter called the community tuberculosis campaign or intervention). The aim of this community tuberculosis campaign was to (i) empower communities to confront tuberculosis; (ii) expand service coverage; and (iii) accelerate progress towards the targets of the End TB Strategy.^15^ The objectives were to improve tuberculosis case identification, management and prevention.

The community tuberculosis campaign in this study also incorporated screening for leprosy. Subsequent rounds which offer tuberculosis, leprosy, HIV, malaria, malnutrition, antenatal care for pregnant mothers, immunization, and COVID-19 services at the community level have been dubbed CAST+. The analysis presented here is limited to the evaluation of campaign 1 and campaign 2 which had a similar scope of diseases (tuberculosis and leprosy).

In this article we present an effectiveness evaluation of two of such community tuberculosis campaigns held in 2022.

## Methods

### Study design

We conducted a quasi-experimental before-and-after study using the RE-AIM framework^16^ to assess the reach, outputs, adoption and effectiveness of the community tuberculosis intervention.

We calculated each as the percentage of achievements compared to set targets. We used four outputs. These outputs were the number of people who were: (i) screened during the campaign; (ii) identified as presumptive tuberculosis patients; (iii) identified with bacteriologically-confirmed tuberculosis and had initiated treatment; and (iv) contacts assessed and initiated on tuberculosis preventive therapy. We measured adoption by the number of districts and implementing partners that embraced the community tuberculosis intervention, and their willingness to integrate it into routine case-finding practices across the country.

To determine effectiveness (impact), we conducted a cross-sectional analysis of bacteriologically-confirmed tuberculosis case notification before (control) and after the community tuberculosis intervention was implemented. For campaign 1 in March 2022, we compared tuberculosis case notifications in the first and second quarters of 2022 with those from the corresponding quarters in 2021 (control). For campaign 2 (September 2022), we compared tuberculosis case notifications in the third and fourth quarters of 2022 with those from the corresponding quarters in 2021 (control). This approach accounts for delayed notifications of tuberculosis cases identified during the intervention month at the end of each quarter, which may only be officially reported in the subsequent quarter.

### Setting

Administratively, Uganda’s 146 districts are divided into 15 health regions. There is regional variation in tuberculosis incidence and notification; the highest case notification rates are in the Karamoja region.^14^

Uganda's decentralized health system is structured with national and regional referral hospitals at the top. These hospitals support general (district) hospitals and health centres (ranging from levels II to IV) and, at the community level, village health teams. Around 1700 of the 6937 public and private facilities are tuberculosis diagnostic and treatment units.

### Intervention description

The community tuberculosis campaign is a biannual, national, community-based, active tuberculosis case-finding initiative which is implemented in March and September of each year. Key components include community mobilization and sensitization; voluntary symptom screening for tuberculosis and testing of presumed individuals for tuberculosis using GeneXpert® (Cepheid, California, United States of America); and linkage to care for those with a confirmed tuberculosis diagnosis. Contacts of peopled diagnosed with tuberculosis are identified and assessed for preventive tuberculosis therapy.

National and district-level planning meetings were held before any activity on the intervention, while mass media was used to raise community awareness.

The tuberculosis programme held a one-day meeting before campaign implementation to orientate village health teams and primary health workers on the package. The programme then assigned each village with a village health team member to operate within their own communities; on average, each member was responsible for visiting 7–15 households per day.

At the start of implementation of the intervention, village health teams introduced themselves and the campaign to household members and requested verbal consent to conduct tuberculosis sensitization and screening. They were equipped with an educational flipchart and one-pager flyers containing basic tuberculosis and leprosy messages, translated into local languages. Every household visited received a flyer.

Village health teams used symptom screening to identify presumptive tuberculosis patients regardless of human immunodeficiency virus (HIV) status: persons with a cough for two weeks or more, or persistent fever, or excessive night sweats or weight loss were identified as presumptive tuberculosis patients.

Village health teams were associated with a tuberculosis diagnostic and treatment unit (hereafter also referred to as “facility”); health workers from these facilities performed contact investigations, targeted screenings in hotspots, tuberculosis diagnosis and treatment initiation.

For contact investigation, health workers first identified index tuberculosis patients whose contacts had not yet been evaluated. They then scheduled appointments with contacts and assessed them for tuberculosis and, where relevant, eligibility for tuberculosis preventive therapy.

Health workers used their facility data to assign hotspots (i.e. areas with the most people with tuberculosis) for targeted community screening. Health workers then coordinated with community leaders for convenient screening times and locations.

All sputum samples collected during screening were analysed using GeneXpert®. Information on confirmed tuberculosis patients was then sent to the facility by a phone call or short message service; the facility in turn relayed the information to the village health teams to link confirmed individuals to treatment at the nearest facility.

Village health team members were given a daily basic allowance of 10 000 Ugandan shillings (around 2.7 United States dollars, [US$]); each health worker received a daily allowance of 12 000 Ugandan shillings (around US$ 3.2) and a transport refund of 30 000 Ugandan shillings (around US$ 7.9).

The community campaign also incorporated screening for leprosy. The village health teams were sensitized on identifying leprosy lesions, and individuals presumed to have leprosy were referred to the health facilities for appropriate evaluation and treatment.

### Implementation process

Campaign 1 and campaign 2 were implemented for 5 days in March and September 2022, respectively. Regions and districts were at liberty to choose a week during March and September 2022 to implement the campaign.

**Fig. 1:**
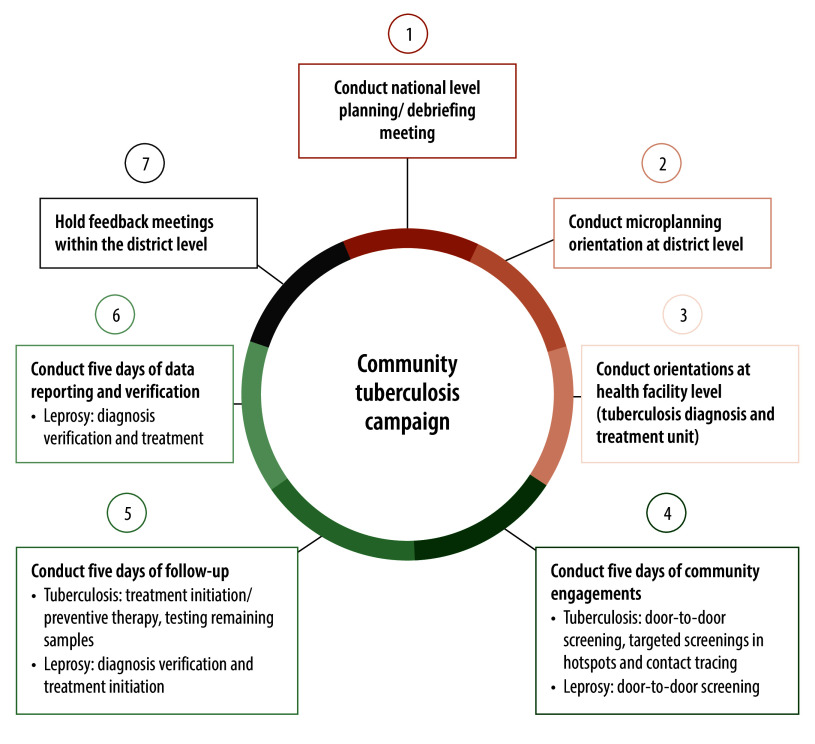
Implementation steps for the community tuberculosis campaign, Uganda, 2021–2022

The community tuberculosis intervention was implemented in seven key steps (Fig. 1). Throughout the campaign, national, district and implementing partner staff provided technical assistance to ensure quality assurance for both facility- and community-level interventions. Participants did not incur any costs.

### Variables

The primary outcome of our analysis was the number of tuberculosis cases notified. This was defined as the number of bacteriologically-confirmed tuberculosis patients diagnosed and notified within the national surveillance system in a quarter.

### Data sources

During the campaign, village health teams used printed forms of the Community TB Sensitization and Screening Register to capture basic information; data were then manually transferred to facility registers. Health workers tallied the specified indicators used to compile facility-level summary reports. Reports were then submitted to the district tuberculosis and leprosy supervisor, verified, and entered into an online system from DEFEAT TB (Kampala, Uganda), by health information assistants and the district biostatistician. The platform has been set up for the tuberculosis programme and partners to monitor and coordinate efforts directed towards defeating tuberculosis in Uganda. The quarterly numbers of tuberculosis patients notified were obtained using District Health Information Software 2 (University of Oslo, Oslo, Norway). We obtained outputs from campaign records such as attendance lists.

### Statistical analysis

We summarized the four outputs of each campaign both nationally and by Uganda’s 15 health regions. 

We compared the total number of bacteriologically-confirmed tuberculosis patients notified and the case notification rate during the eligible community tuberculosis campaign quarters of the previous year (before the campaign) with that of the corresponding eligible quarters during the campaign. Tallies of specified tuberculosis and leprosy indicators from the online system are presented in text, tables and figures.

We used the Stata *csi* command (StataCorp, College Station, USA) to estimate the case notification rate and case notification rate ratio from the number of tuberculosis cases notified in each period based on projected populations. 

We also compared tuberculosis notifications during the quarters that the intervention took place in participating districts with notifications for corresponding quarters in non-participating (control) districts (further information is available in the authors’ online repository).^17^ Again, we used the *csi* command to estimate the case notification rates and rate ratios. We present our results according to the reporting guidelines for implementation and operational research.^18^

### Ethical considerations

Ethical approval for the study was obtained from Mengo Hospital Research Ethics Committee (MH-2023–30). Screening, testing and treatment for tuberculosis and the provision of tuberculosis preventive therapy to community members was voluntary.

## Results

We present the targets and achievements of the two campaign cycles in Table 1. In campaign 1, 111 out of 146 districts (76%) conducted district planning and orientation meetings. A total of 643 of all 1700 diagnostic and treatment units in the country (37.8%) trained and orientated village health teams. Campaign 1 screened a total of 1 289 213/44 212 800 people (2.9% of the Ugandan population), while campaign 2 screened 5 134 056 people (11.6% of the Ugandan population).

**Table 1:** Pre-implementation targets and reach, by campaign cycles, Uganda, 2021–2022

Step	Campaign 1 (Mar 2022)			Campaign 2 (Sep 2022)	
	Targets, no.	Reach, no. (%)		Targets, no.	Reach, no. (%)
National planning meetings	1	1 (100.0)		1	1 (100.0)
District planning and orientation meetings	146	111 (76.0)		146	146 (100.0)
Planning and orientation meetings by tuberculosis diagnostic and treatment unit	1 700	643 (37.8)		1700	1 608 (94.6)
Individual screenings	44 212 800	1 289 213 (2.9)		44 212 800	5 134 056 (11.6)
**Districts implementing the campaign, by region**					
Acholi	9	5 (55.6)		9	9 (100.0)
Ankole	13	9 (69.2)		13	13 (100.0)
Bugisu	10	2 (20.0)		10	10 (100.0)
Bukedi	7	4 (57.1)		7	7 (100.0)
Bunyoro	9	9 (100.0)		9	9 (100.0)
Busoga	12	11 (91.7)		12	12 (100.0)
Kampala	1	1 (100.0)		1	1 (100.0)
Karamoja	9	9 (100.0)		9	9 (100.0)
Kigezi	6	6 (100.0)		6	6 (100.0)
Lango	10	10 (100.0)		10	10 (100.0)
North Central	12	10 (83.3)		12	12 (100.0)
South Central	14	11 (78.6)		14	14 (100.0)
Teso	11	12 (109.1)		11	11 (100.0)
Tooro	10	5 (50.0)		10	10 (100.0)
West Nile	13	13 (100.0)		13	13 (100.0)
**Total**	**146**	**117 (80.1)**		**146**	**146 (100.0)**

Of the 1 289 213 screened in campaign 1, a total of 179 144 (13.9%) were identified as presumptive tuberculosis patients. The proportion of those screened that were identified as presumptive tuberculosis patients differed by region; this proportion was highest in Bukedi (2703 out of 6955; 38.9%) and lowest in the West Nile region (7161 out of 106 216; 6.7%). Of all the presumptive tuberculosis cases in campaign 1, 4043 people (2.3%) were diagnosed with bacteriologically-confirmed tuberculosis. By region, Bunyoro had the highest proportion (451 out of 4821; 9.4%) of presumptive tuberculosis patients diagnosed with tuberculosis. Of the total of 4043 people whose diagnosis of tuberculosis was confirmed, 3710 (91.8%) initiated treatment. Five regions had treatment initiation rates below 90% (Ankole, Karamoja, Kigezi, Teso and West Nile) with losses-to-follow-up before treatment that ranged from 17.4% to 38.0% (100% minus percentage initiating treatment), while three regions (Bunyoro, North Central and Tooro) reported higher proportions of individuals who initiated tuberculosis treatment during the campaigns. In campaign 1, 8024 people received tuberculosis preventive therapy (Table 2).

**Table 2:** Comparison of campaign 1 and 2, overall and by region, Uganda, 2021–2022

Region	Campaign 1 (Mar 2022)						Campaign 2 (Sep 2022)				
	Screened, no.	Presumptive cases, no. (%)	Sample collected, no. (%)	Confirmed cases, no. (%)	Treatment initiated, no. (%)		Screened, no.	Presumptive cases, no. (%)	Sample collected, no. (%)	Confirmed cases, no. (%)	Treatment initiated, no. (%)
Acholi	157 422	11 002 (7.0)	11 896 (108.1)	254 (2.3)	229 (90.2)		291 863	31 836 (10.9)	26 039 (81.7)	555 (1.7)	400 (72.1)
Ankole	175 794	18 273 (10.4)	16 028 (87.7)	307 (1.7)	238 (77.5)		650 180	28 317 (4.4)	23 576 (83.2)	488 (1.7)	474 (97.1)
Bugisu	22 372	3 974 (17.8)	1 885 (47.4)	139 (3.5)	132 (95.0)		447 713	30 297 (6.8)	21 817 (72)	490 (1.6)	461 (94.1)
Bukedi	6 955	2 703 (38.9)	1 853 (68.5)	102 (3.8)	96 (94.1)		181 381	29 224 (16.1)	16 374 (56)	257 (0.9)	245 (95.3)
Bunyoro	21 999	4 821 (21.9)	4 806 (99.6)	451 (9.4)	451 (100)^b^		190 527	19 665 (10.3)	13 960 (70.9)	286 (1.5)	275 (96.2)
Busoga	91 610	17 527 (19.1)	11 534 (65.8)	607 (3.5)	583 (96.0)		386 068	29 283 (7.6)	17 023 (58.1)	436 (1.5)	433 (99.3)
Kampala	4 985	1 682 (33.7)	1 361 (80.9)	43 (2.5)	42 (97.7)		37 685	6760 (17.9)	4820 (71.3)	136 (2.0)	127 (93.4)
Karamoja	83 173	14 308 (17.2)	5 648 (39.4)	195 (1.4)	161 (82.6)		167 637	20 815 (12.4)	18 695 (89.8)	391 (1.9)	383 (98)
Kigezi	25 975	5 936 (22.9)	5 446 (91.7)	79 (1.3)	49 (62.0)		210 893	18 109 (8.6)	13 471 (74.3)	151 (0.8)	149 (98.7)
Lango	159 370	34 319 (21.5)	27 185 (79.2)	475 (1.4)	457 (96.2)		293 265	54 804 (18.7)	21 728 (39.6)	556 (1.0)	474 (85.3)
North Central	68 561	13 551 (19.8)	8 610 (63.5)	316 (2.3)	316 (100)^b^		296 177	28 290 (9.6)	17 170 (60.6)	735 (2.3)	421 (57.3)
South Central	50 475	7 377 (14.6)	5 367 (72.7)	275 (3.7)	268 (97.5)		112 891	15 582 (13.8)	13 259 (85)	642 (4.1)	387 (60.3)
Teso	242 505	29 364 (12.1)	22 709 (77.3)	277 (0.9)	207 (74.7)		532 936	17 510 (3.3)	14 880 (84.9)	245 (1.4)	238 (97.1)
Tooro	71 801	7 146 (10.0)	4 999 (69.9)	299 (4.2)	299 (100)^b^		354 822	31 923 (9.0)	24 635 (77.1)	712 (2.2)	416 (58.4)
West Nile	106 216	7 161 (6.7)	5 268 (73.5)	224 (3.1)	182 (81.3)		980 018	66 029 (6.7)	50 085 (75.8)	1 064 (1.6)	1 059 (99.5)
**Overall**	**1 289 213**	**179 144 (13.9)**	**134 595 (75.1)**	**4 043 (2.3)**	**3 710 (91.8)**		**5 134 056**	**428 444 (8.3)**	**297 532 (69.4)**	**7 144 (1.7)**	**5 942 (83.2)**

^a^ Confirmed bacteriologically.

^b^ Proportion of people linked to care was higher than 100% (Bunyoro 452/451; 100.2%; North Central 380/316; 120.3%; and Tooro 308/299; 103%). The proportion of people linked to care was capped at 100% in these districts to avoid biasing the overall linkage to care proportion upwards.

In campaign 2, all 146 districts (100%) and 1608 of 1700 (94.6%) diagnostic and treatment units conducted relevant orientation meetings. A total of 71 564 village health teams participated in campaign 2, screening an average of 30 households per village health team (Table 1). 

Out of the 5 134 056 people screened in campaign 2, 428 444 (8.3%) were identified as presumptive tuberculosis patients and of these, 7144 (1.7%) were confirmed with tuberculosis (Table 2). By region, the proportion of presumptive tuberculosis patients among the screened population ranged from 3.3% to 18.7%. Of those diagnosed with tuberculosis, 5942 individuals (83.2%) were started on treatment. The treatment initiation rates in different regions ranged from 57.3% to 99.5%. During campaign 2, 23 547 people received tuberculosis preventive therapy.

Compared to hotspot screening and contact tracing, more cases were identified from the door-to-door screening (3410 for campaign 1 and 5969 cases for campaign 2), with village health teams visiting 615 438 households in campaign 1, and 2 174 595 households in campaign 2.

Of the 134 595 sputum samples collected during campaign 1, 117 975 (87.7%) were analysed; while of the 297 532 samples collected during campaign 2, 225 813 (75.9%) were analysed.

Campaign 1 identified 39 patients with leprosy of whom 22 (56.4%) initiated treatment; while campaign 2 identified 137 patients of whom 115 (83.9%) initiated treatment (further information is available in the authors’ online repository).^17^

### Intervention impact 

**Fig. 2:**
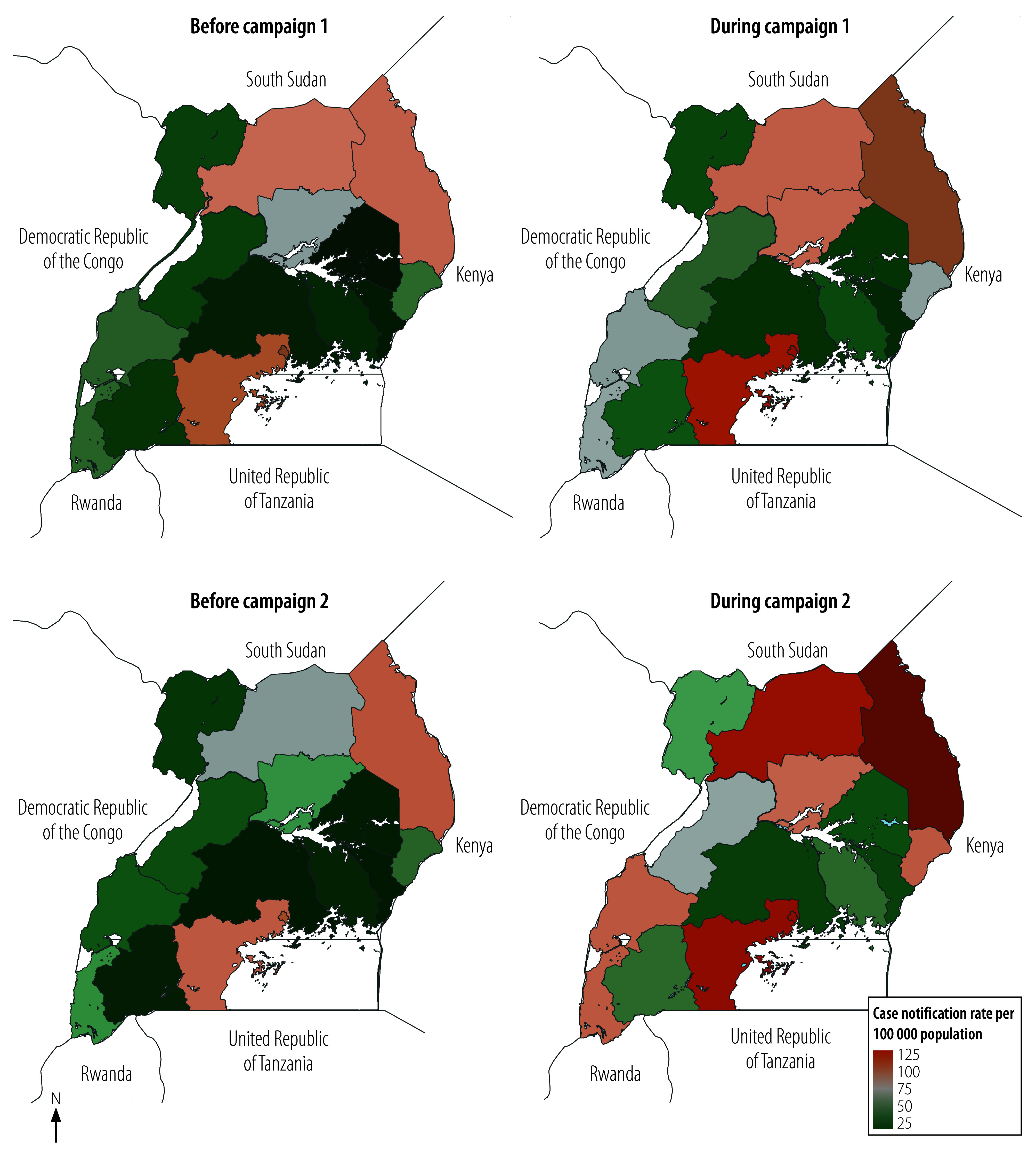
Tuberculosis case notification rates before and during two community campaigns, Uganda, 2021–2022

In Fig. 2, we show maps of case notification rates by region before and during community tuberculosis campaign 1 and campaign 2. 

In our before-and-after analysis, improvements in case notification rates with campaign implementation were observed for both campaign cycles 1 and 2. For campaign 1, the case notification rate increased from 48.1 per 100 000 population before the campaign to 59.5 per 100 000 during the campaign (Table 3); the case notification rate ratio was 1.24 (95% CI: 1.22–1.26). For campaign 2, the case notification rate increased from 45.0 per 100 000 population to 71.6 per 100 000; the case notification rate ratio was 1.59 (95% CI: 1.56–1.62). 

**Table 3:** Comparison of bacteriologically-confirmed tuberculosis case notifications (case notification rates), by campaign cycle overall and region before and after the intervention, Uganda, 2021–2022

Region	Population	Campaign 1				Campaign 2		
		No. of cases (case notification rate)^a^		Case notification rate ratio (95% CI)^b^		No. of cases (case notification rate)^a^		Case notification rate ratio (95% CI)^b^
		Before	During			Before	During	
Acholi	1 474 300	1 247 (84.6)	1 299 (88.1)	1.04 (0.96–1.13)		1 073 (72.8)	1 794 (121.7)	1.67 (1.55–1.80)
Ankole	4 072 500	1 504 (36.9)	1 985 (48.7)	1.32 (1.23–1.41)		1 160 (28.5)	2 328 (57.2)	2.01 (1.87–2.15)
Bugisu	1 536 700	883 (57.5)	1 168 (76.0)	1.32 (1.21–1.44)		847 (55.1)	1 405 (91.4)	1.66 (1.52–1.81)
Bukedi	2 714 100	728 (26.8)	873 (32.2)	1.20 (1.09–1.32)		729 (26.9)	1 143 (42.1)	1.57 (1.43–1.72)
Bunyoro	2 885 500	1 186 (41.1)	1 561 (54.1)	1.32 (1.22–1.42)		1 374 (47.6)	2 083 (72.2)	1.52 (1.42–1.62)
Busoga	4 866 100	1 595 (32.8)	2 188 (45.0)	1.37 (1.29–1.46)		1 492 (30.7)	2 749 (56.5)	1.84 (1.73–1.96)
Kampala	1 738 600	1 842 (105.9)	2 142 (123.2)	1.16 (1.09–1.24)		1 679 (96.6)	2 214 (127.3)	1.32 (1.24–1.40)
Karamoja	1 245 600	1 087 (87.3)	1 315 (105.6)	1.21 (1.12–1.31)		1 174 (94.3)	1 679 (134.8)	1.43 (1.33–1.54)
Kigezi	899 600	498 (55.4)	649 (72.1)	1.30 (1.16–1.46)		527 (58.6)	817 (90.8)	1.55 (1.39–1.73)
Lango	2 583 100	1 818 (70.4)	2 259 (87.5)	1.24 (1.17–1.32)		1 577 (61.1)	2 226 (86.2)	1.41 (1.32–1.51)
North Central	7 928 000	2 297 (29.0)	2 706 (34.1)	1.18 (1.11–1.25)		2 113 (26.7)	3 297 (41.6)	1.56 (1.48–1.65)
South Central	2 686 500	2 626 (97.7)	3 127 (116.4)	1.19 (1.13–1.25)		2 400 (89.3)	3 402 (126.6)	1.42 (1.35–1.49)
Teso	2 607 000	577 (22.1)	967 (37.1)	1.68 (1.51–1.86)		726 (27.8)	1 197 (45.9)	1.65 (1.50–1.81)
Tooro	3 366 700	1 821 (54.1)	2 462 (73.1)	1.35 (1.27–1.44)		1 652 (49.1)	3 038 (90.2)	1.84 (1.73–1.95)
West Nile	3 608 500	1 540 (42.7)	1 592 (44.1)	1.03 (0.96–1.11)		1 381 (38.3)	2 306 (63.9)	1.67 (1.56–1.78)
**Overall**	**44 212 800**	**21 249 (48.1)**	**26 293 (59.5)**	**1.24 (1.22–1.26)**		**19 904 (45.0)**	**31 678 (71.6)**	**1.59 (1.56–1.62)**

^a^ Per 100 000 population.

^b^ Considering 2021 as baseline.

In an inter-district comparison for campaign 1, districts that implemented the intervention had a higher case notification rate than districts that did not implement a campaign, at 51.6 and 49.0 per 100 000 population, respectively.^17^ The case notification rate ratio was 1.05 (95% CI: 1.01–1.10).

## Discussion

We set out to evaluate the impact of expansive community tuberculosis screening in two community tuberculosis campaigns. Both campaigns successfully reached a substantial percentage of the targeted populations. Overall, both campaigns identified a notable proportion of individuals with presumed tuberculosis (13.9% and 8.3%, respectively), although there were some regional variations; in the absence of the intervention, such community members might not have sought care for their symptoms. Campaign 1 and 2 also resulted in increased case notification rates for bacteriologically-confirmed tuberculosis.

According to current World Health Organization guidelines, systematic screening for tuberculosis at the population level is recommended in areas where the tuberculosis prevalence is at least 5000 cases per 100 000 population.^19^ As it allows for intermittent community screening, the community tuberculosis campaign used in this study offers an alternative approach for high-burden countries that do not meet the threshold for systematic screening.

The community tuberculosis campaign described here was largely modelled on the DETECTB trial in Zimbabwe, which compared two twice-yearly community tuberculosis active case finding interventions that used either mobile vans or door-to-door visits.^20^ The community campaign in this study used symptom screening during door-to-door visits, hotspot screening and contact tracing; the diagnostic yields observed underscore the large pool of undiagnosed tuberculosis within the communities.^21^ The slightly lower yield observed during campaign 2 could partly be attributed to campaign 1 having treated some cases of tuberculosis in the community.^20^ A previous systematic review of diagnostic yields from active case finding strategies showed variability in yields that were dependent on the population and tuberculosis screening and diagnostic approach.^22^ Although the presumptive and diagnostic yields were generally considerable, the substantial variation across the regions highlights the need for programme improvement by implementing tailored interventions, including refining the screening methods in regions with low presumptive and diagnostic yields.

The campaigns very likely contributed to the notable increase in the case notification rate for bacteriologically-confirmed tuberculosis because of the intervention. This observed increase in case notification rate contrasts with previous evidence suggesting that community-based tuberculosis screening did not consistently increase the case notification rate.^11^ That finding, however, could have been a consequence of using smear microscopy and chest radiographs for tuberculosis diagnosis. In another campaign, with no observed impact on the case notification rate, researchers attributed findings to rapid decline in tuberculosis burden.^23^

A major challenge that we experienced during implementation of the community tuberculosis intervention was delay in the initiation of tuberculosis treatment; this delay resulted in high levels of pre-treatment loss-to-follow-up, particularly during campaign 2. These pre-treatment losses are a concern in all community tuberculosis case finding interventions as highlighted by previous studies.^24^^,^^25^ Integrating patient-centred care approaches into community tuberculosis case finding interventions is vital to mitigate various provider and patient factors that influence losses.^26^ Notably, tuberculosis diagnostic and treatment units received overwhelming sample volumes that exceeded their processing capacity despite continuous machine operation. Recurrent breakdowns of GeneXpert® machines or cartridge shortages prompted the redistribution of samples to alternative GeneXpert® sites for analysis and, in some regions like West Nile, necessitated staggered implementation of the campaign.

Our study has some limitations. First, we did not assess the treatment outcomes of the tuberculosis patients diagnosed during the intervention. Second, we also did not assess the economic and social benefits of the community tuberculosis intervention to families and households, and did not assess the impact of the intervention on tuberculosis epidemiology in communities. Third, analysis of data by age group and gender was not performed as data were not collected in a case-based format; this limited extended disaggregated analysis. We recommend that future studies prioritize these aspects for investigation. Further studies will evaluate the feasibility and cost-effectiveness of the intervention.

In conclusion, our study highlights the potential of community-based tuberculosis case finding campaigns in accelerating progress towards targets and goals of the End TB Strategy through early detection and treatment of undiagnosed tuberculosis cases within communities. However, continuous monitoring and adaptation, including tailored interventions to overcome context specific implementation challenges, must be included in future campaigns.
